# Cerebral cortex and autonomic nervous system responses during emotional memory processing

**DOI:** 10.1371/journal.pone.0229890

**Published:** 2020-03-05

**Authors:** Yuko Mizuno-Matsumoto, Yuji Inoguchi, Steven M. A. Carpels, Ayumi Muramatsu, Yusuke Yamamoto

**Affiliations:** Graduate School of Applied Informatics, University of Hyogo, Kobe, Japan; Waseda University, JAPAN

## Abstract

Psychiatric symptoms are often accompanied by somatic symptoms induced by the activity of the autonomic nervous system (ANS). The aim of this study was to calculate the time lag between electroencephalography (EEG) and electrocardiography (ECG) responses, to clarify the changes in the relationship between the cerebral cortex (CC) and the sympathetic nervous system (SNS) during emotional recall processing. Twenty-two healthy young adults were examined. Their EEG and ECG data were simultaneously recorded during emotional audiovisual recall tasks using pleasant and unpleasant stimuli for 180 s, with three repetitions (Epochs 1 & 2 and Epoch 3). The EEG data were analyzed using a fast Fourier transform (FFT) to obtain a time series of relative power spectra, *X*_*E*_, in the theta 1, theta 2, alpha 1, alpha 2, alpha 3, beta 1, beta 2, and beta 3 bands. Time series of *RR* (inter-beat) intervals (time intervals between successive R waves) derived from the ECG spectral analysis using FFT was applied to the resampled time series of *RR* intervals over about 60 s to obtain a time series of power spectra for the ratio low frequency/high frequency (LH/HF), *X*_*C*_, which reflects the activity of the sympathetic nervous function. The time lag between *X*_*E*_ and *X*_*C*_ was calculated using wavelet-crosscorrelation analysis. The results demonstrated that the brain responded to unfamiliar emotionally pleasant stimuli in Epochs 1 & 2 earlier than the SNS, whereas the brain and SNS responded to unfamiliar unpleasant stimuli nearly simultaneously. The brain was activated rapidly in response to familiar unpleasant stimuli, although SNS responded more rapidly to familiar pleasant stimuli than the brain in Epoch 3. Our results quantitatively describe the relationship between the CC and the ANS during emotional recall.

## Introduction

The intimate connection between the brain and the heart was enunciated by Claude Bernard [1]. The heart is influenced by brain activity. The brain can bidirectionally interact with the heart via the sympathetic and parasympathetic (vagus) nerves. Heart rate variability (HRV) plays an important role in the emotional regulation of body functions to adapt to constantly changing environmental conditions.

Psychiatric symptoms are often accompanied by somatic symptoms induced by the activity of the autonomic nervous system (ANS) [2]. Stimulation of the ANS causes cardiovascular, muscular, gastrointestinal, and respiratory symptoms (e.g., diarrhea, dizziness, hypertension, palpitations, or tremor). The cingulate gyrus (an element of the Papez circuit) is a part of the central nervous system that plays a key role in emotional processes. The cingulate gyrus also influences the hypothalamus, which is involved in the regulation of the ANS. This hypothalamic involvement in the ANS implicates psychosomatic disorders and mental disorders with somatic symptoms.

It is important to understand how mental stress and emotional stimuli cause reactions in the brain and the ANS, and how both interact in the processing of emotions. It has been reported that recall of happiness is correlated with parasympathetic activity, and right frontal activation during recall of happiness is associated with a decrease in sympathetic activity [3]. Using a public speech preparation task in a functional magnetic resonance imaging (fMRI) study, it was found that cortical activity mediates heart rate (HR) responses to social evaluative threat [4]. It was also demonstrated that during sleep, brain rhythms in the electroencephalography (EEG) and the HRs in the electrocardiography (ECG) are coordinated in time and exhibit characteristic time delays [5].

Several studies of brain responses to emotional stimuli were recently published [6–8]. Studies using EEG found that in healthy participants, the frontal cortex is activated by emotional events [9,10]. We reported that the EEG responses of the cerebral cortex (CC) to unpleasant emotional stimuli are different from the responses to pleasant emotional stimuli [11–13]. We also evaluated the responses of the ANS to pleasant and unpleasant emotional stimuli using ECG and plethysmography [13,14].

In most of the previous studies, the CC response and ANS response have been examined separately. However, it is essential to reveal the interaction of brain and heart functions as a first step towards comprehending the total reaction of the whole human body in an emotional situation. The medical engineering technique proposed in this study could prove helpful for psychiatric medical treatments in the future.

In our previous research, we showed that simultaneous recordings of EEGs and ECGs are helpful to clarify the relationship between the brain and the ANS [15]. We examined time lag (TL) changes between EEG and activation of the parasympathetic nervous system in response to emotional memory recall [16]. The ANS consists of sympathetic and parasympathetic nerves, which often but not always exert antagonistic functions [17]. In the present study, we focused on the sympathetic nervous system (SNS) to complement our previous findings in the parasympathetic nervous system, thus comprehensively elucidating ANS reactions.

Here, we examined brain and SNS parameters based on correlations between EEG and ECG recordings, which are electrical time-series data. These data could be helpful in clarifying how emotional stimuli affect brain and ANS activities and reveal time-dependent interactions between these structures. The objective of this study was to evaluate the correlations between brain oscillatory activity and HRV during emotional recall using TL analysis of EEGs and ECGs. These findings are novel and significant in the fields of neuroscience, physiology, and medical engineering.

## Materials and methods

### Study participants

Twenty-two healthy young adults (mean age ± SD [standard deviation] 24.7 ± 4.6 years, 7 women and 15 men) participated in this study. The study protocol was approved by the Ethics Committee of the Graduate School of Applied Informatics, University of Hyogo (H-20-001). All participants gave written informed consent after receiving a detailed explanation of the experimental purposes and research protocol. The study was conducted in accordance with the 1964 Declaration of Helsinki and its later amendments of comparable ethical standards.

### EEG & ECG recordings and data acquisition

EEG and ECG recordings were simultaneously performed using a Nihon-Kohden Inc. system (Tokyo, Japan) with 19 EEG electrodes positioned according to the international 10/20 system (Fp1, Fp2, F3, F4, C3, C4, P3, P4, O1, O2, F7, F8, T3, T4, T5, T6, Fz, Cz, Pz) and with ECG electrodes placed on the right wrist and the left ankle (Lead II). The EEG and ECG data were digitized at a sampling rate of 500 Hz and filtered offline between 0.5 and 50 Hz.

**Fig 1:**
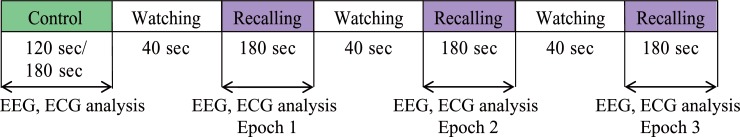
Experimental protocol. The emotional audiovisual recall task required the participants to watch a video footage for 40 seconds (Watching) and recall the contents of the videos with their eyes closed for 180 seconds (Recalling). The participants were presented with pleasant stimuli in session 1 and with unpleasant stimuli in session 2. In the beginning of each session, a quiescent state with the eyes closed was imposed (Control) for 120 seconds in session 1 or 180 seconds in session 2. This sequential watching-and-recalling epoch was repeated 3 times (epochs 1, 2, and 3) with the same kind of stimuli. EEG and ECG were analyzed during the Control and Recall periods. The subjects were instructed to breathe naturally during EEG and ECG recordings.

EEG and ECG were recorded during emotional audiovisual recall tasks using two different conditions: pleasant and unpleasant sessions. The participants were presented with pleasant audiovisual stimuli, such as a comedy video in session 1, and with unpleasant audiovisual stimuli such as a horror movie in session 2. The order of the two sessions was fixed in all participants. Prior studies confirmed that the order effect is negligible [15]. In a preliminary experiment, the positive and negative impressions from all audiovisual stimuli were assessed using a visual analog scale (VAS). The stimuli with high positive and negative scores in the preliminary experiment were adopted as pleasant and unpleasant stimuli in this experiment, respectively. The participants were asked to watch video footages as emotional stimuli for 40 s (“Watching” in Fig 1) and recall the contents with their eyes closed for 180 s (“Recalling” in Fig 1). The sequence of watching and recalling was repeated three times (Epochs 1, 2, and 3) with the same kind of stimuli (pleasant stimuli in session 1, and unpleasant stimuli in session 2). The stimuli in epochs 1 and 2 were supposed to be “unfamiliar stimuli” for the participants, whereas the stimuli in epoch 3 were considered as “familiar stimuli.” At the beginning of each session, a quiescent period with eyes closed was conducted as the Control (120 s in session 1 and 180 s in session 2) (Fig 1). The Control in session 1 was shorter to save time for the participants. The experimental protocol was designed according to our previous research [11], to adopt a non-randomization block-wise presentation. EEG and ECG recordings during recall of the movie contents in Epochs 1, 2, and 3 and during the Control period were analyzed.

### EEG analysis

**Fig 2:**
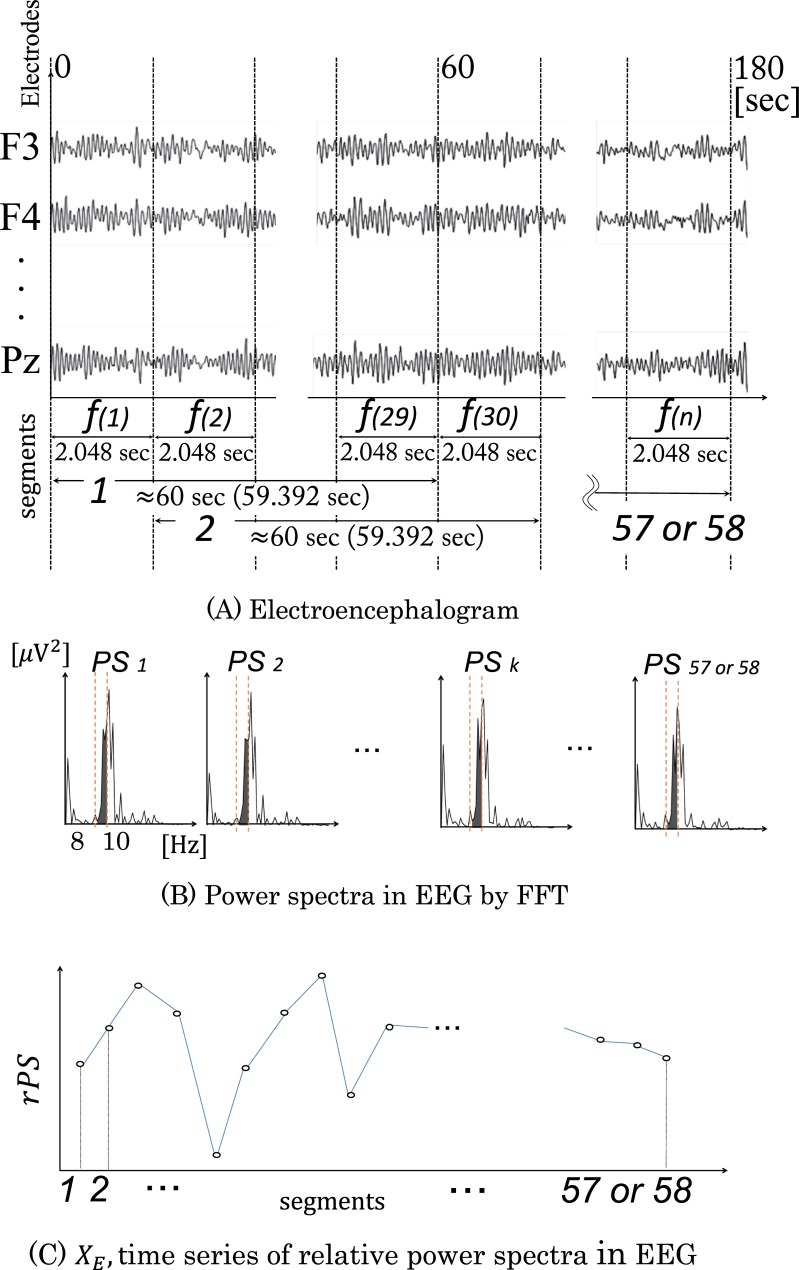
Procedure of the EEG analysis. (A) EEG data were analyzed using a fast Fourier transform to obtain power spectra every 2.048 s. (B) Twenty-nine power spectra (59.392 s) were averaged to obtain mean power spectra *PS*_*k*_ in all the 19 electrodes. (C) The power spectra were normalized by the power spectra values from the Control period to get relative power spectrum density ratios (*rPS*). We then calculated the relative power spectra (*rPS*) in the theta 1 (4–6 Hz), theta 2 (6–8 Hz), alpha 1 (8–10 Hz), alpha 2 (10–12 Hz), alpha 3 (12–14 Hz), beta 1 (14–16 Hz), beta 2 (16–18 Hz), and beta 3 (18–20 Hz) bands, in fifty-seven (114.688–174.080 s) or fifty-eight (116.736–176.128 s) segments, to obtain a time series of relative EEG power spectra (*X*_*E*_).

Fig 2 shows the procedure for the EEG analysis. EEG data ((*f(n)* in Fig 2(A)) were analyzed using a fast Fourier transform (FFT) to obtain power spectra every 2.048 s. Twenty-nine power spectra values (about 60 s) were averaged to get mean power spectra *PS*_*k*_ (Fig 2(B)) for all 19 electrodes. The mean power spectra *PS*_*k*_ were normalized to the power spectra values from the Control period, to get relative power spectrum density ratios *rPS* (Fig 2(C)). We calculated the relative power spectra *rPS* in the theta 1 (4–6 Hz), theta 2 (6–8 Hz), alpha 1 (8–10 Hz), alpha 2 (10–12 Hz), alpha 3 (12–14 Hz), beta 1 (14–16 Hz), beta 2 (16–18 Hz), and beta 3 (18–20 Hz) bands in 57 (114.688–174.080 s) or 58 (116.736–176.128 s) segments, to obtain a time series of relative EEG power spectra in all epochs. *X*_*E*_ was defined as a time series of relative EEG power spectra (Fig 2(C)). Although the length of the raw EEG data was 180 s, artifact-free EEG data (57 or 58 segments) was used for analysis.

About 2-s long segments were used for the EEG analysis in agreement with our previous research [11,18]. About 60-s long segments were established to calculate the mean power spectra values from the values determined in 2-s long segments because the shortest acceptable length for the ECG analysis (described in the next section) was 60 s [19].

### ECG analysis

**Fig 3:**
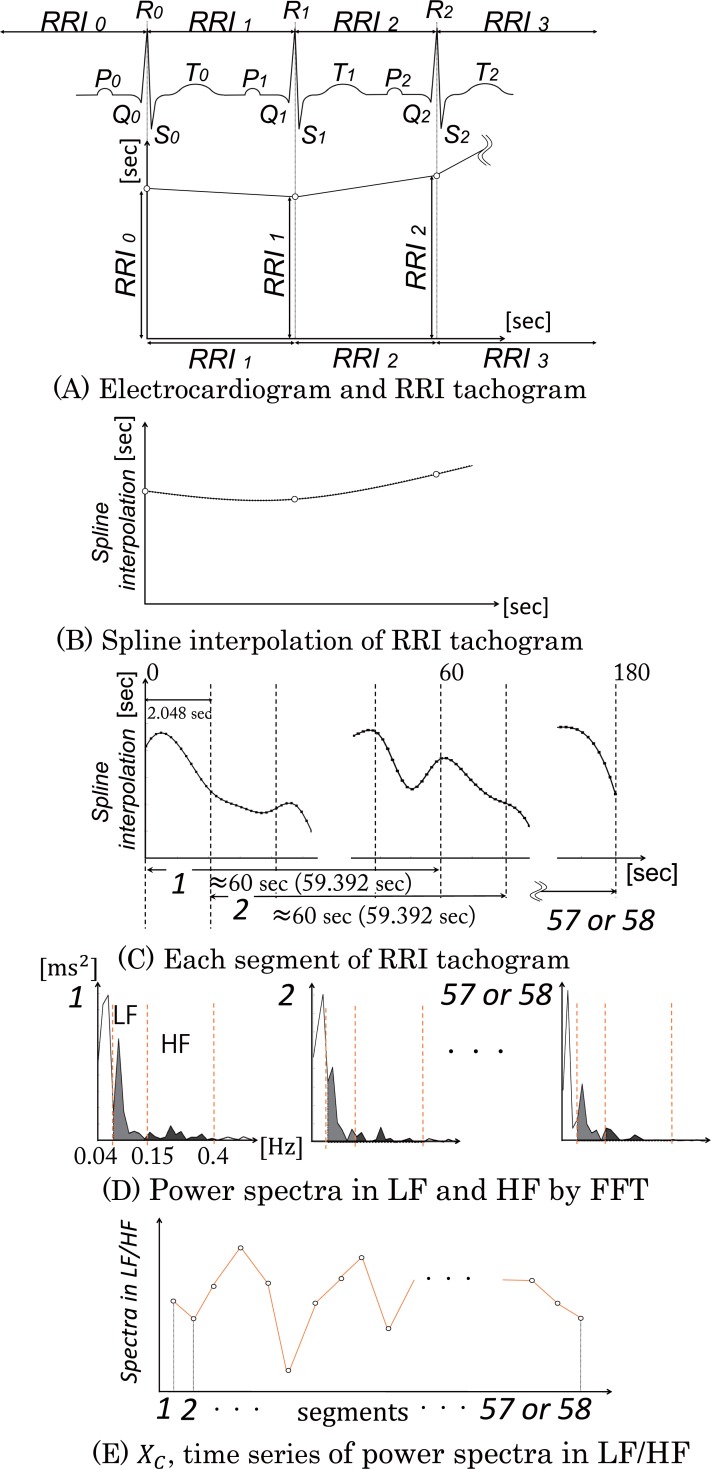
Procedure of the ECG analysis. (A) A series of RR intervals (*RRI*_*n*_) was derived from the ECG. (B) After the spline interpolation, resampling was conducted. (C) Spectral analysis using a fast Fourier transform was applied to the resampled time series of the RR interval for about 60 s (59.392 s) at intervals of 2.048 s. (D) The absolute power spectra values in low frequency (LF, 0.04–0.15 Hz) and high frequency (HF, 0.15–0.40 Hz) components were obtained. (E) The power spectra of LF/HF were calculated from the power spectra values of LF and HF in fifty-seven or fifty-eight segments to obtain a time series of the power spectra of LF/HF (*X*_*C*_).

Fig 3 shows the procedure for the ECG analysis, which was used to quantify HRVs. A series of *RR* intervals (*RRI*_*n*_ in Fig 3(A), the time interval between successive R waves) was derived from the ECG. After resampling in 6.25 Hz using spline interpolation (Fig 3(B)), spectral analysis using FFT was applied to the resampled time series of *RR* intervals for about 60 s (59.392 s) at intervals of 2.048 s (Fig 3(C)). The absolute power spectra of low frequency (LF; 0.04–0.15 Hz) and high frequency (HF; 0.15–0.40 Hz) components were obtained (Fig 3(D)). The power spectra values of the ratio of LF to HF (LF/HF) were calculated from the spectra values of LF and HF in 57 or 58 segments to obtain their time series. *X*_*C*_ was defined as the time series of the LF/HF power spectra (Fig 3(E)). The LF/HF spectra values are considered to reflect the activity of the sympathovagal balance [20].

### Correlation analysis between EEG and ECG

A correlation analysis between *X*_*E*_ from an EEG and *X*_*C*_ from an ECG was conducted using the wavelet-crosscorrelation analysis (WCA) [18,21,22]. Values for the wavelet-crosscorrelation coefficient (WCC) and the TL between two non-stationary time series were obtained using the WCA.

The WCC value, *WR*_*x*,*y*_(*a*,*τ*) from the real part of the wavelet-crosscorrelation function is

$${WR}_{x,y}(a,\tau )=\frac{{RWC}_{x,y}(a,\tau )}{\sqrt{{RWC}_{x}(a,0){RWC}_{y}(a,0)}}$$ (1)

where *a* is a scale parameter and τ is a time delay of the wavelet coefficients in the wavelet space. The wavelet-crosscorrelation function *WR*_*x*,*y*_(*a*,*τ*) is complex-valued, and *RWC*_*x*,*y*_(*a*,*τ*) is the real part of the function for two signals *x* and *y*. The TL *τ*_max *x*,*y*,*a*_ that gives the maximum WCC is

$$\tau_{\max\;x,y,a}=argmax\;{WR}_{x,y}(a,\tau ),(-L_{a}\leq \tau \leq L_{a})$$ (2)

where *La* corresponds to the half-length of one wave for each scale *a*.

In this study, TLs between *X*_*E*_ from EEG recordings of each electrode and *X*_*C*_ from the ECG were only calculated if WCC was greater than or equal to the threshold value 0.8 [15,16]. As an exception, if all WCC values in a subject were less than 0.8, a threshold of 0.7 or 0.75 was used for this subject’s analysis. We adopted the WCC threshold according to our previous research [21]. The mean TL values *mτ*_*EC*_ were calculated over Epochs 1 and 2 and separately over Epoch 3. Since the values of Epochs 1 and 2 were always similar, only their summary is provided in the Results section. *X*_*E*_ precedes *X*_*C*_ if the value for *mτ*_*EC*_ is greater than zero, whereas *X*_*C*_ precedes *X*_*E*_ if the value for *mτ*_*EC*_ is less than zero. Only if *mτ*_*EC*_ is zero, *X*_*E*_ and *X*_*C*_ appear simultaneously. In the time sequences *X*_*E*_ and *X*_*C*_, 2.048 s correspond to one “point”. The same unit “point” is also employed to describe the mean TL values derived from the differences between the time sequences *X*_*E*_ and *X*_*C*_, as well as the unit in figures. For visualization of the results, the mean TL values *mτ*_*EC*_ are shown in color for each electrode, for each frequency band, for pleasant and unpleasant stimuli, as well as for Epochs 1 & 2 and Epoch 3.

### Statistical analysis

The mean TL values were statistically evaluated using three methodologies.

**(1) Comparison between pleasant and unpleasant stimuli for each electrode.** The mean TL values *mτ*_*EC*_ across all participants were statistically compared between pleasant and unpleasant stimuli for each electrode in each frequency band in Epochs 1 & 2 and Epoch 3 using the paired t-test. *P* values were adjusted for multiple testing and a false discover rate (FDR)<0.05 was considered statistically significant.

**(2) Evaluation of whether the absolute value is larger than zero or not for each electrode.** The mean TL value *mτ*_*EC*_ for each electrode across all participants was statistically compared with the comparison value (zero in this study) for pleasant and unpleasant stimuli in each frequency band in Epochs 1 & 2 and Epoch 3 using the one sample t-test.

**(3) Comparison between pleasant and unpleasant stimuli across all electrodes.** The mean TL values *mτ*_*EC*_ across all electrodes and all participants were statistically compared between pleasant and unpleasant stimuli in each frequency band in Epochs 1 & 2 and Epoch 3 using the paired t-test. *P* values were adjusted for multiple testing, and a false discover rate (FDR)<0.05, 0.01, or 0.001 was considered statistically significant.

## Results

**Fig 4:**
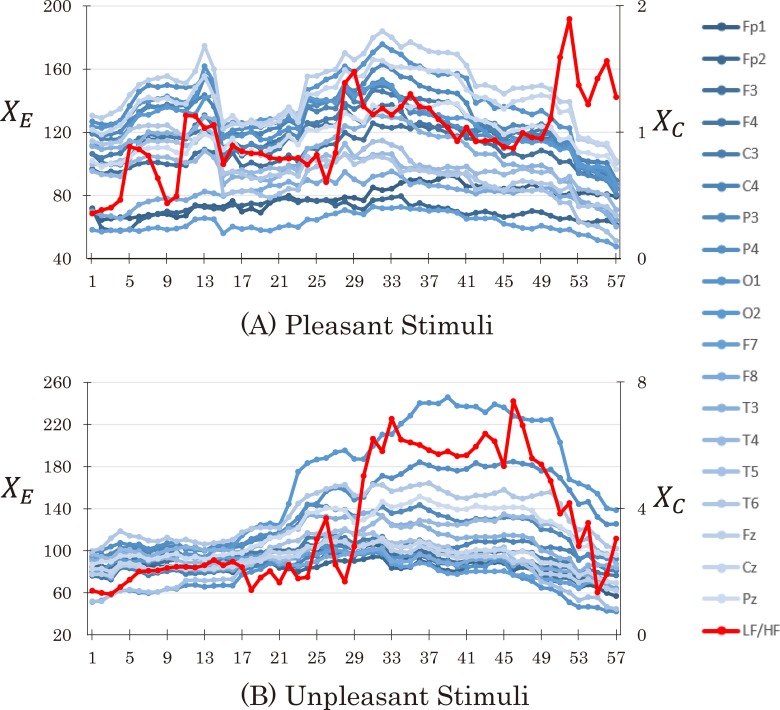
An example of *X*_*E*_ and *X*_*C*_.

Fig 4 shows an example of *X*_*E*_ and *X*_*C*_ values in an epoch for pleasant (A) and unpleasant (B) stimuli in epoch 1. The blue lines indicate *X*_*E*_ values derived from recordings at different EEG electrodes, and the red line displays *X*_*C*_ derived from the LF/HF ratio of the ECG.

**Fig 5:**
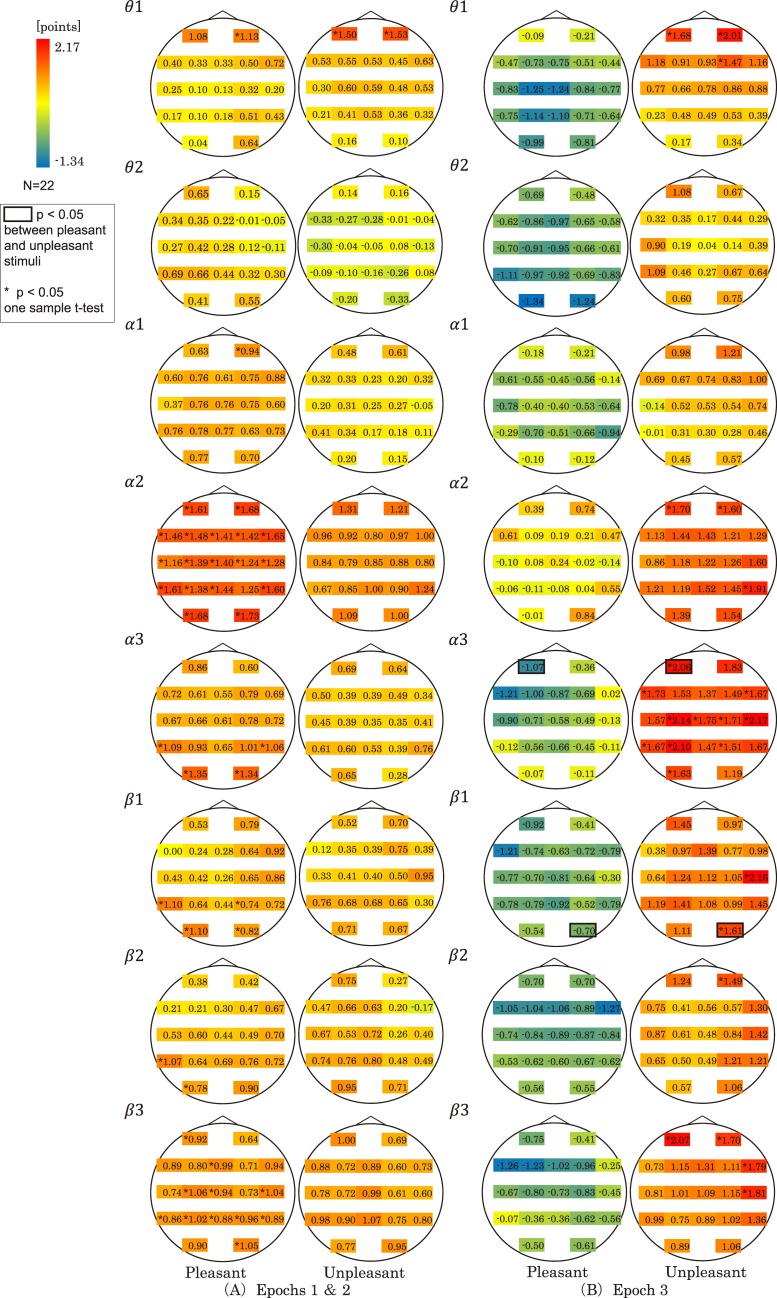
Map of mean TL values. (A) Mean TL values across all participants for pleasant and unpleasant stimuli for each frequency band (*θ*1,*θ*2,*α*1,*α2*, *α3*,*β*1,*β*2,*β*3) in Epochs 1 and 2. The color range is fixed in the maps for pleasant and unpleasant stimuli. When the mean TL value *mτ*_*EC*_ is larger than zero, this means that *X*_*E*_ precedes *X*_*C*_. When *mτ*_*EC*_ is smaller than zero, this means that *X*_*C*_ precedes *X*_*E*_. When *mτ*_*EC*_ is zero, this means that *X*_*E*_ and *X*_*C*_ appear simultaneously. One point of mean TL value between the time sequences *X*_*E*_ and *X*_*C*_ corresponds to 2.048 s. (B) Mean TL values in Epoch 3.

Fig 5 maps with a fixed color range across all electrodes the mean TL values across all participants for pleasant and unpleasant stimuli in each frequency band in Epochs 1 & 2 (Fig 5(A)) and Epoch 3 (Fig 5(B)). Black boxes indicate significant differences between mean TL values of pleasant and unpleasant stimuli on the same electrode. Asterisks show significant differences between mean TL values of each electrode and the comparison value (zero) using the one sample t-test.

Fig 5(A) demonstrates that in Epochs 1 & 2, the mean TL values for almost all electrodes in alpha 2 and beta 3 bands, for Fp2 in theta 1 and alpha 1 bands, for T5, O1, O2, and T6 in alpha 3 band, for T5, O1, P4, and O2 in beta 1 band, and T5 and O1 in beta 2 band for pleasant stimuli were significantly greater than zero (* in Fig 5). For unpleasant stimuli, however, there was no significant difference between mean TL value and zero for all the electrodes, except for Fp1 and Fp2 in theta 1 band.

Fig 5(B) shows that in Epoch3, there was no significant difference between the mean TL values for pleasant stimuli and zero for all the electrodes in all frequency bands. However, the mean TL values for almost all electrodes in alpha 3 band, for Fp1, Fp2, and F4 in theta 1 band, for Fp1, Fp2, and T6 in alpha 2 band, for T4 and O2 in beta 1 band, for Fp2 in beta 2 band, and for Fp1, Fp2, F8, and T4 in beta 3 band were significantly larger than zero (* in Fig 5).

In Fig 5(A) and 5(B), no significant differences between the mean TL values of pleasant and unpleasant stimuli on the same electrodes were observed in Epochs 1 & 2. In Epoch3, however, there were significant differences between the mean TL values of pleasant and unpleasant stimuli for Fp1 in alpha 3 and for O2 in beta 1 bands (black squares in Fig 5).

**Fig 6:**
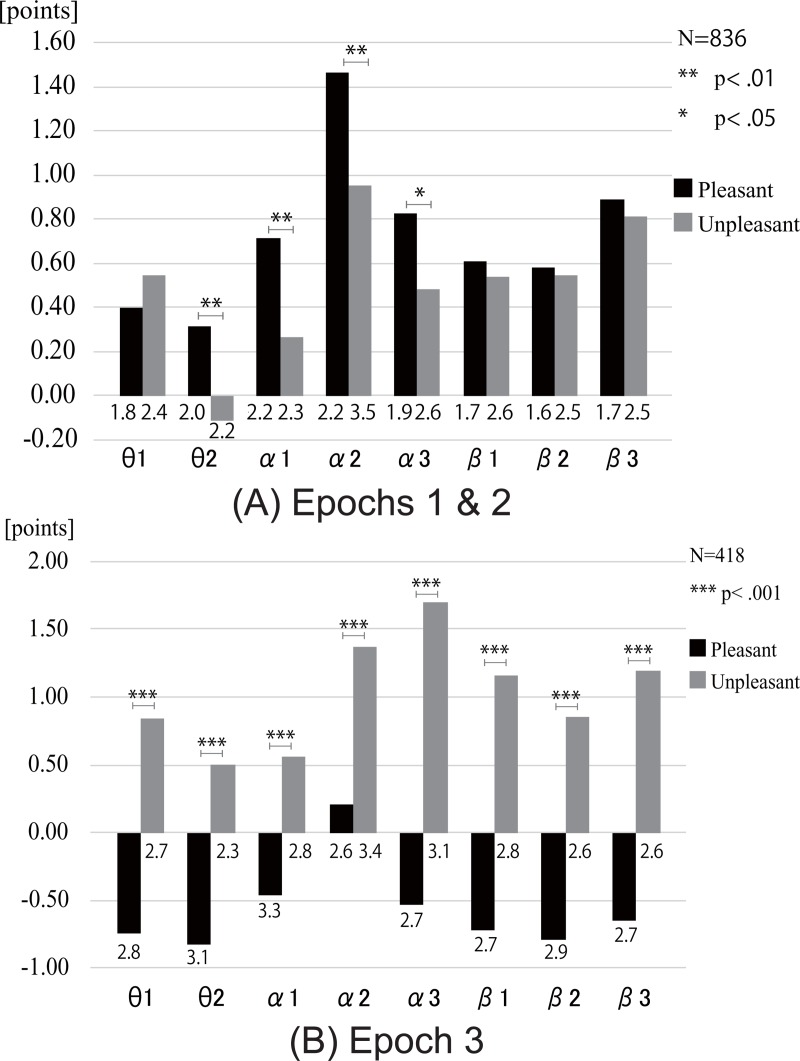
Mean TL values. (A) Mean TL values across all electrodes and all participants for the pleasant and unpleasant stimuli in each frequency band in Epochs 1 and 2. Black bars show the results for pleasant stimuli, and gray bars show the results for unpleasant stimuli. The numbers below the bars represent the standard deviations. (B) Mean TL values across all electrodes and all participants for the pleasant and unpleasant stimuli in each frequency band in Epoch 3.

Fig 6(A) shows the mean TL values across all electrodes and all participants for pleasant and unpleasant stimuli in each frequency band in Epochs 1 & 2. Black and gray bars indicate the results for pleasant and unpleasant stimuli, respectively. The numbers below the bars represent the standard deviations. These results demonstrate that the mean TL values for pleasant stimuli are greater than those for unpleasant stimuli in the theta 2, alpha 1, alpha 2, and alpha 3 bands.

Fig 6(B) presents the mean TL values across all electrodes and all participants, for pleasant and unpleasant stimuli in each frequency band in Epoch 3. The mean TL values for unpleasant stimuli are significantly greater than those for pleasant stimuli in all the frequency bands.

## Discussion

Our results demonstrate that in Epochs 1 & 2, the brain responded faster to unfamiliar emotionally pleasant stimuli compared to the SNS, although the brain and SNS responded to unfamiliar unpleasant stimuli almost simultaneously. In Epoch 3, the SNS displayed earlier than the brain signs of activation in response to familiar pleasant stimuli, whereas familiar unpleasant stimuli evoked brain activity prior to sympathetic nervous activity.

Our research is based on the idea that there is an intimate connection between the brain and the heart, as advocated by Claude Bernard [1]. The response patterns of the brain and the ANS should be different according to whether the emotional stimuli are pleasant or unpleasant, and whether the emotional stimuli are familiar or unfamiliar. Using EEG and ECG recordings, we characterized the response speeds of the CC and the ANS for familiar or unfamiliar, pleasant or unpleasant stimuli.

Thayer et al. showed that the prefrontal cortex tonically inhibits cardioacceleratory circuits [23]. The prefrontal cortex has extensive connections with other cortical and subcortical regions, including the hypothalamus, and regulates thoughts, actions, and emotions [24]. The prefrontal cortex is also an important part of the circuitry that implements both positive and negative affects [25,26]. Thus, the medial prefrontal cortex could have specific roles in emotional decision-making and emotional self-regulation [27].

The medial temporal lobe includes the amygdala, which helps to focus declarative memory for complex stimuli encoded under emotional contexts through its differential effects on memory. The amygdala modulates the hippocampus, affecting memory for gist [28]. Despite the fact that the hippocampal-frontal circuitry is important for spatial and temporal contexts, the circuitry is particularly sensitive to stress. It has been reported that acute stress causes a remarkable and long-lasting inhibition of long-term potentiation at synapses in the frontal cortex evoked by stimulation of the hippocampal outflow [29]. As such, plasticity at hippocampal to prefrontal cortex synapses can be affected by stress.

Association fibers interconnect various areas of the cortex within the same brain hemisphere in longitudinal directions, whereas commissural fibers connect an area in one hemisphere with an area in the opposite hemisphere in coronal directions. The activities in frontal, central, parietal, and temporal areas can propagate in longitudinal and coronal directions according to the activated fibers.

Theta band oscillations in frontal regions are associated with theta activity in the hippocampus [30]. Memory associated with emotion can be related to theta activity, and theta activity increases between the amygdala and the hippocampus after fear conditioning. Alpha band activity also changes during memory operations [30]. There have been reports of enhanced prefrontal alpha amplitude in a short-term memory task. Alpha enhancement is frequently observed during mental imagery, imagination, and internal attention [30]. The upper alpha band exhibits reactivity to the presence of semantic memory and increases with memory demands [30]. The beta activity appears when participants are emotionally unstable, not attentive to their internal thinking, or in excited states. Aversive visual stimulation evokes an increase in frontal fast-beta power [31].

The reported findings and our new results indicate that emotional stimuli provoke functional activity in the prefrontal cortex to discriminate whether a stimulus is harmful. The prefrontal cortex regulates the hypothalamus via the cingulate cortex to activate or inactivate the ANS. When stimuli are unpleasant, information could pass through a fast or short circuit from the prefrontal area to the hypothalamus [1]. Therefore, the response of the SNS to unpleasant stimuli is faster than the response to pleasant stimuli. The fast activity of the brain and the ANS could allow quick judgment and motor behavior in harmful situations. However, when emotional stimuli have already been familiar and are pleasant, the prefrontal cortex response is slow, while the ANS responds automatically in the same way as for unfamiliar stimuli. By contrast, even if emotional stimuli already have been familiar, unpleasant stimuli can provoke large responses in the CC to assess whether the stimuli are harmful, making use of emotional memory in the hippocampus and amygdala. Moreover, the early CC responses in the fast frequency bands of the EEG to familiar unpleasant stimuli may indicate an aversive memory process. Our current results also indicate that CC activity can propagate between frontal, central, parietal, and temporal areas. The results showed the interconnectivity between various areas of the CC.

In this research, the standard deviations of TL values in Fig 6 were large. This event shows that the inter-individual variability could exist, when the mean TL values were calculated. We need to aware that the inter-individual variability could be included in the generalizability findings. In this current experiment, we did not measure respiratory rhythm. It was necessary to measure respiratory rhythm to assure that the rhythm was within HF (0.15–0.4Hz) range and the depth of the respiration did not differ among the different conditions.

In this study, a negative TL value signifies that the CC responds after the SNS. This event could be caused by two mechanisms: either the CC responds slowly or the SNS responds more rapidly than the CC. The negative TL value for familiar pleasant stimuli may have occurred due to the former reason. Physiological responses to an affective situation can be indicative of habituation. The CC may respond to pleasant stimuli rapidly when the stimuli are unfamiliar, whereas the CC may respond to pleasant stimuli slowly when the stimuli are familiar. By contrast, the SNS could respond to pleasant stimuli in the same way, irrespective of whether the stimuli have been familiar before or not.

## Conclusions

We analyzed EEG and ECG recordings after emotional stimuli to reveal the TL between the responses of the CC and the ANS. We found that the brain responded faster than the ANS to unfamiliar emotionally pleasant stimuli, whereas the brain and ANS responded to unfamiliar unpleasant stimuli nearly simultaneously. ANS activation to unfamiliar, unpleasant stimuli was more rapid than that to pleasant stimuli. The brain responded more rapidly to familiar unpleasant stimuli than the ANS, although it did not respond rapidly to familiar pleasant stimuli. The CC may regulate the hypothalamus to activate or inactivate the ANS according to whether the external information is unpleasant or pleasant and unfamiliar or familiar. Our results quantitatively describe the relationship between the CC and the ANS during emotional recall.
